# COVID-19 and women's nutrition security: panel data evidence from rural India

**DOI:** 10.1007/s40888-021-00233-9

**Published:** 2021-07-02

**Authors:** Soumya Gupta, Payal Seth, Mathew Abraham, Prabhu Pingali

**Affiliations:** Tata-Cornell Institute for Agriculture and Nutrition, Cornell University, Ithaca, USA

**Keywords:** Diet diversity, Food security, Nutrition, COVID-19, Rural markets, Women, I14, I15, J16

## Abstract

In response to the COVID-19 pandemic, India implemented a stringent nationwide lockdown. Although food value chains and allied activities were exempted from the lockdown, there were widespread disruptions in food access and availability. Using two panel-datasets, we distinguish the pandemic's impact on non-staples versus staples in relation to household food availability and women’s diet diversity at the national, state, and district levels in four economically backward districts of Uttar Pradesh (Maharajganj), Bihar (Munger), and Odisha (Kandhamal and Kalahandi). Both the primary and secondary data indicate a decline in household food expenditures and women’s dietary diversity in May 2020 compared to May 2019, particularly for non-staples like meats, eggs, vegetables and fruits. This occurred despite special PDS, direct benefit transfer, and ration from *aanganwadis* rations reaching 80%, 50%, and 30% of surveyed households, respectively. While national and state-level expenditures recovered to the pre-lockdown levels by June 2020, the district-level expenditures did not recover. Our findings contribute to the growing body of evidence of women's disproportionate vulnerability to economic shocks, the impact of a staple grain focused safety net program, and restricted markets on the access and availability of diverse nutritious foods. This paper makes a case for policy reforms towards PDS diversification to include nutrition-rich foods and market reforms to remove supply-side bottlenecks and expansion of direct benefit transfers for healthy food access. We also highlight the importance of gender-responsive safety nets and their increased coverage for improving intrahousehold nutritional disadvantages.

## Introduction

Global pandemics can severely affect food and nutrition security in developing countries, exacerbating the already existing malnutrition burdens (Brinkman et al., 2010; Padhee & Pingali, 2020; Tiwari & Zaman, 2010). The COVID-19 pandemic is expected to have an enormous impact on economic growth and development and food and nutrition security, disproportionately affecting marginal social groups, women, and children (Akseer et al., 2020; Swinnen & McDermott, 2020). As governments respond to the pandemic with lockdowns, supply and demand-side factors such as inflation, income losses, informal food market disruptions, and the inaccessibility of government safety net programs were expected to worsen food security, especially for the poor (Devereux et al., 2020; Headey & Ruel, 2020). Supply bottlenecks increased the prices of perishable, micronutrient-rich nonstaples more than staple foods such as grains and pulses (Laborde et al., 2020), resulting in a shift in consumption away from vegetables, meats, and dairy, towards cheaper calorie-dense cereals (Headey & Ruel, 2020). Evidence from phone surveys from various countries and locations such as rural China and urban Ethiopia support this evidence (Hirvonen et al., 2020; Rozelle et al., 2020; Tesfaye et al., 2020). This study focuses on assessing the pandemic impact on women's nutrition security in four districts in Uttar Pradesh, Bihar, and Odisha. Relying on a primary and secondary panel of data on food expenditures and women’s diet diversity, we distinguish the impact on staples versus non-staples. This distinction is crucial as an increase in calorie deficit—arising from reduced consumption of staples—is distinct from a worsening quality of diets that affects food security and, more importantly, nutrition security (Rahman et al., 2020; Seth et al., 2020).

After the WHO declared COVID-19 as a global pandemic, India enforced a stringent nationwide lockdown beginning on March 24, 2020, restricting people's movement and transportation of non-essential goods all services. Although food value chains and allied activities were exempted from the lockdown, there were widespread disruptions in supply chains resulting from labor shortages, credit freezes, and cross-border travel restrictions and movement of goods (Abhishek et al., 2020). Price data collected from various urban centers indicate a disproportionate increase in nonstaples prices compared to staple foods (Seth et al., 2020) resulting from these disruptions. Declining incomes from loss of employment opportunities and food price inflation meant that an estimated 8–32 million Indians were at risk of not being able to afford nutritious food in the initial 21 days of the lockdown (Gupta & Madgavkar, 2020).

Household-level food access and availability are adversely affected by the loss of income and breakdown of food markets, and disruption in food supply from in-kind safety net programs (Laborde et al., 2020). But periods of crisis can highlight the urgent need for reform as they test the resilience of markets and welfare programs and form the impetus for long-term policy reforms (Padhee & Pingali, 2020). The lockdown did bring the shortcomings of existing safety net programs to the forefront and the need for a more market-oriented resilience of food systems. First, the Public Distribution System that provides subsidized staples to 800 million citizens provided extra grains for free. However, they lacked diversity, especially concerning nutrition-rich foods, potentially impacting vulnerable groups such as women and children. Second, the mid-day meal program that provides hot cooked meals to primary school children and *aanganwadi* centers serving preschool children and pregnant and lactating mothers with supplementary nutrition programs were most affected as schools and *aanganwadi* centers closed. To improve the access and availability of diverse, nutritious foods, direct benefit transfers through which households can assess healthy foods from markets become relevant. The Indian government introduced cash transfers of $22 billion and expanding the initiative to $279 billion by May 2020 (Dev, 2020), and these need to be expanded and established in post-pandemic periods. Well-functioning and deregulated markets also become critical to ensure effective price discovery, reduce supply constraints, and ensure nutritious food availability and affordability to vulnerable groups.

### Objective of the study

This paper uses two panel datasets to assess how food and nutrition security indicators were impacted by an exogenous shock like the COVID-19 pandemic. The two research questions this study asks are:

- What is the pandemic's impact on household food availability at the national and subnational level in India?
- What impact did the pandemic have on women's food access and diet diversity at the intra-household level?

Using the Consumer Pyramids Household Survey (CPHS), a nationally stratified panel dataset on state and district-level food expenditure, we assess changes in household food expenditure in May–June 2020 compared to May 2019. Changes in food expenditures are analyzed at three levels: national, state (Bihar, Odisha and Uttar Pradesh) and district (Munger, Maharajganj, Kandhamal and Kalahandi). At each geographical unit, the expenditures are disaggregated in order to distinguish the association that the pandemic had on spending on staples versus non-staple food groups. A similar analysis of changes in household food expenditures is undertaken using primary data from a phone survey that was implemented in May 2020 with a May 2019 baseline. Using the same dataset, we also extend the analysis to look at the differential impact of the pandemic on women's consumption via changes in their dietary diversity and the potential influence of access to safety net programs on food availability.

### Household food security-disentangling issues of access to nutritious foods and disproportionate impact on women

Shocks such as the COVID-19 pandemic and nationwide lockdowns can affect access and availability of food in two ways, first, through the loss of income and reducing individual-level food access, lower dietary diversity, and a switch to low-quality diets (Darmon & Drewnowski, 2015; UN, 2020). Second, the disruption of value chains leads to inflation-induced price rise for nearly all food groups, accentuating access problems when coupled with income loss. However, the changes in dietary quality are unlikely to be distributed evenly across the household. It is presumed that the pandemic will have different implications on women's food and nutrition security than other household members based on the role that women play in agriculture, employment, food purchase and preparation, and the structural barriers they face in these domains (Doss et al., 2020). Therefore, considering the impact shocks have on food availability, diversity of food, and the differential effects on vulnerable groups, especially women, are critical in emergency responses and overall policy reforms for food security resilience.

Safety nets in the form of cash transfer and food-based safety net programs can safeguard household vulnerability to income and availability shocks. Food safety net programs that provide non-perishables like rice, wheat, and pulses, instead of a diversified basket of nonstaples will further reinforce monotonous diets lacking crucial micronutrients (Headey & Ruel, 2020). However, the elasticity of demand for nutrient-rich foods being much higher than that for staple grains—direct benefit transfers in the form of conditional or unconditional cash transfers can improve access to nutritious foods (Almås et al., 2019). Disentangling the change in food access from PDS and cash transfer programs can shed light on the nutritional quality of food households consumed during the pandemic. Two studies try to capture the extent of safety net program outreach during the lockdown. Kesar et al. (2020) in their phone survey carried on rural and urban workers across 12 states of India, observe that household access to safety net relief measures was not equal. While nearly 80% of households had received the PDS ration, anywhere between 40 and 60% did not receive any cash transfers under various government schemes. In his phone survey, Acharya (2020) shows that just about one-third of the eligible households received take-home ration as of May 2020 and the Mid-Day Meal Scheme's reach being even less. Cash transfer and in-kind food provisions will have a disparate impact on household-level food security regarding the quantity and quality of foods.

### Women's nutrition status and intrahousehold food allocation

Health outcomes for women and girls will be disproportionately affected by increasing scarcity of food, patriarchal social norms influencing intrahousehold food allocation (de Paz et al., 2020), and workloads that influence the time allocated to nutritious food preparation (Doss et al., 2020). The gender disadvantage is also supported by evidence that income and price elasticity of food intake varies by gender and age. Global income and price elasticities of food demand for women and young girls are greater than those for men and young boys, particularly for fruits and vegetables (Muhammad et al., 2017). The trend is especially worrisome since the consumption of fresh fruits and vegetables is the first nutrition advice propagated by WHO for adult diets during the COVID-19 pandemic (WHO, 2020). Lower consumption of fruits and vegetables is expected to exacerbate the already prevalent micronutrient deficiencies in rural India, especially among children and women (Gonmei & Toteja, 2018), putting them at a greater risk of infection mortality (McAuliffe et al., 2020).

One of the primary risk factors for maternal and child undernutrition in the current pandemic is low-quality diets resulting from loss of incomes, supply chain disruptions, and halting of government food safety nets (Akseer et al., 2020). In India, where 53% of women (15–49 years) and nearly 60% of children under-five were anemic (IIPS, 2017) before the pandemic. As per the 2019–2020 NFHS-5 data, the rates of under-5 child stunting and anemia in women of reproductive ages (15–49 years) in rural Bihar were 43.9% and 63.1%, respectively. Some of these health outcomes, like anemia, worsened in 2019–2020 compared to 2015–2016. Although little is known about how the pandemic will impact women's health and access to food, it could accentuate existing disadvantages. The data suggest a worsening of intra-household food distribution for two reasons. First, we know that women's diets are lacking in nutrient-dense foods. The NFHS-4 data (IIPS, 2017) indicates that just about 30% of women consume fruits, meats, and fish weekly in India. The corresponding figure for the consumption of eggs and dark green leafy vegetables is just under 40%. Second, and more importantly, women's consumption rates are lower than that for men in these food groups. In rural India, the proportion of women who consumed meats and eggs lag behind men by nearly ten percentage points, while that for fruits and fish lags by six percentage points (IIPS, 2017). More recently, evidence from rural UP, Bihar, and Odisha show that women faced a dietary gap even before the pandemic, wherein their dietary diversity lags behind that of other members of their households. Gupta et al. (2020) have shown that of the households where households where non-staples like fruits, vegetables, meats and dairy were consumed, such foods were consumed by the woman in approximately 20% or fewer of those households. There is evidence that the pandemic has had a gendered impact on nutrition security in India. Harris et al. (2020) in their survey of male and female farmers across Assam, Jharkhand, AP and Karnataka find that diets of female farmers were more adversely affected as compared to male farmers. Both, the affordability and consumption of vegetables, fruits and dairy products was significantly reduced amongst women farmers as compared to male farmers.

To assess the disproportionate impact on women and expenditure changes at the national, state, and district levels, we collected quantitative data and qualitative self-reported data from various households to understand the household and the intra-household level changes during the lockdown. While assessing the impact of the lockdown on food security at the national, women's inherent disadvantages in access to nutritious foods make it imperative to look at the implication for women's nutrition, as they will be disproportionately impacted. The study also differentiates access of staples and nonstaples at the state, district, and household level. While we look at the availability of different food groups, we focus on women's consumption and diet diversity. We were thus able to determine the impact the lockdown had on the quality of food consumed at the household level and the specific effects on women's diet diversity.

## Data, methods, and measurement

The study uses two sets of panel data. The CPHS is a national-level panel of secondary data on household food expenditures maintained by the Centre for Monitoring of the Indian Economy (CMIE). We look at food expenditures at three levels—national, state and district—for March–June 2019 and 2020. Additionally, we use primary data collected at the district-level during May 2020 (phone survey) with corresponding baseline surveys for the same households in May 2019 (in-person survey). This dataset provides details about food consumption changes at the household level and dietary diversity among women. The two measurements deployed in the survey are the food access and affordability indicator and a women's dietary diversity indicator.

Some rapid phone-based surveys assess India's emerging food security conditions following the lockdown pointed to worsening food security. Phone-based surveys are becoming increasingly popular to collect data, especially with the proliferation of mobile phone use (Gibson et al., 2017). While there are several limitations, including not reaching areas with low connectivity and households without cellphones, the surveys are cost-effective and can be rolled out in much shorter time frames, making it practical for rapid surveys. In Ceballos et al., (2020) phone survey in Harayana and Odisha, households reported inability to access or afford sufficient quantity and variety of foods. Kesar et al. (2020) phone survey of 12 states, 75% of respondents reported consuming less food than before the lockdown, and 35% not having enough money to buy a week’s worth of essential goods like food, water, and healthcare. Acharya (2020) phone study concludes that between 50 and 60% of households in the different states faced shortages of food items (mostly nonstaples such as fresh foods and vegetables) in the 30 days preceding the survey and reduced food intake during the lockdown. The limitations of previous studies using telephone surveys were that they (a) were primarily qualitative using self-reported measures to determine food security outcomes (Acharya, 2020; Ceballos et al., 2020; Kesar et al., 2020); (b) the survey data collected were cross-sectional, without a reliable baseline for comparison and assess the extent of change first; (c) they did not distinguish between to access and consumption of staples and nonstaples separately and (d) except for Acharya (2020), focused on the household at large not accounting for intrahousehold food allocation or the disproportionate effect on women. The following section details the study's location, data collection, analytical framework, validation, and measurement.

### Secondary panel-data on food expenditures

We analyze food demand changes at the household level using secondary data from the Consumer Pyramids Household Survey (CPHS). The CPHS is a national-level panel dataset maintained by the Centre for Monitoring of the Indian Economy (CMIE) detailing consumption expenditure from both rural and urban areas at the district-level. The survey uses a multi-stage stratified survey design, ensuring an equal chance of selecting subgroups and allowing inferences to be made on the sample population. The CPHS surveys households three times a year to capture changes to incomes (called Income Pyramids) and expenditures (called Consumption Pyramids). We focused on CPHS data at three levels: national, state (UP, Bihar, Odisha) and district level (Maharajganj (UP), Munger (Bihar), and Kandhamal and Kalahandi (Odisha)) for the periods between March and June 2019 and 2020. These districts are selected as they are also the sites where primary data—in May 2019 and 2020-was collected (described in the next section). By looking at the national and state-levels, in addition to district-level expenditures, we are able to identify subnational heterogeneity in how economic access to food changed after the pandemic onset.

The CPHS does not have data for Munger district in Bihar. Munger is a part of the homogenous region of Darbhanga–Bhagalpur with similar agroclimatic conditions, levels of urbanization, female literacy, and household size. We consider average CPHS data for the Muger-bordering districts of Begusarai, Bhagalpur, and Khagaria as a substitute for Munger. The consumption pyramids collect detailed, disaggregated household expenditure data for a total of 153 food and non-food items. For the study, we use expenditures on the following food groups: cereals, pulses, fruits, vegetables, milk and milk products, dry fruits, meat and fish, eggs, potatoes and onions and other foods (that includes butter/ ghee, processed/ packaged foods and beverages). Expenditures are analyzed at the national, state and district-level and disaggregated by the food groups listed above.

### District-level primary panel-data

The four districts selected for the phone survey were Munger (Bihar), Maharajganj (Uttar Pradesh), and Kandhamal and Kalahandi (Odisha). The locations are among the most economically backward districts in some of the high malnutrition burden states. The districts were also the site of the Technical Assistance and Research in Indian Nutrition and Agriculture (TARINA) program, a Tata-Cornell Institute for Agriculture and Nutrition, Cornell University-led study centered around designing and promoting nutrition-sensitive food systems in India. The program has been working in four districts since 2015. In May of 2019, TARINA collected data on women's dietary diversity and household food expenditures, amongst other agriculture-nutrition indicators, as part of its midline survey covering 3600 households in these four program districts. Following the lockdown, we surveyed a sub-set of households—using a phone survey—to assess nutrition indicators' changes using the May 2019 survey as the baseline. We reason that data from households in these districts can reveal how the most marginalized regions and populations fared during the pandemic.

The telephone survey sampled 155 households from the 3600 households surveyed under that TARINA midline (May 2019) in May 2020. The survey by telephone helped remote data collection without traveling to the location and served as a rapid assessment tool. The study intended to assess the effect of the COVID-19 pandemic, an exogenous shock on household-level food security in rural India. All the surveyed households were rural, low-income, small, and marginal agricultural households expected to be especially vulnerable to food insecurity from shocks. The phone survey focused on three aspects of food security—(a) food access and affordability from rural markets, (b) food availability at the household level, and (c) food consumption at the individual level. The survey comprised of questions related to women's consumption of various food groups in the past 24 h, households' expenditures on different food groups, coping strategies during the lockdown, loss of earning, access to the market for food purchase, and households' self-reported measures related to food availability, adequacy in quantity and variety. Additionally, the survey asked questions about different benefits received from the government welfare schemes and safety net programs. The phone survey allowed us to determine household food security and women's diet quality during the lockdown period (May 2020) and compare it to the TARINA midline survey (May 2019), matched at the household level. The panel data gives us the unique advantage of quantifying the precise shutdown impact magnitude through an effective baseline. Quantifying this impact is crucial to design future mitigation policy efforts that can help overcome the short and medium-term adverse health effects.

### Measurement of food and nutrition security indicators

This study's two primary measures are food access and affordability and Women's Dietary Diversity Scores (WDDS). As rural agricultural households are both net producers and consumers of food, the food access, and affordability measures capture households' access to food from markets and government programs. The WDDS are constructed based on women's access to diverse foods that potentially determine their nutritional status.

#### Food access and affordability

Rural households rely on food markets to meet their consumption needs, especially for nutrient-dense, non-perishables like fruits, vegetables, and meat products. In contrast, staples (such as rice and wheat) and pulses are grown at the farm-level for household consumption and can be accessed at subsidized rates through food safety programs such as the public distribution system (PDS). In our survey design account for food access from two sources: markets and government-programs. At the household level, the primary surveys in May 2019 and 2020 asked if the household purchased any of the following food groups: grains, pulses, nuts, dairy, meat–fish–poultry (MFP), eggs; dark green leafy vegetables or DGLV; other vegetables, and other fruits. The 2020 survey used a 7-day recall to collect data on total food expenditure in the past one week and a disaggregated food expenditure for each of the food groups mentioned above. The 2019 survey used a monthly recall for expenditure data. Household expenditures were converted to weekly expenditures (dividing by a factor of four) to make them comparable to the 155 household data collected in May 2020. Expenditure on the above-mentioned food groups for May 2020 is compared with May 2019. Although we adjust monthly expenditures to make them comparable to weekly estimates, we note that they are not directly comparable. This is because while a weekly recall reflects recent food purchases, it is likely going to under-report expenditure on those food items that are purchased less frequently by the household. For instance, if meats are purchased once a month then it is more likely to be captured in a monthly recall as opposed to a 7-day recall. A monthly recall will likely reflect variations in food purchases across weeks.

We also analyzed changes to household monthly food expenditures using data from the CPHS at the national, state and district levels. Changes are examined month-to-month for the period May–June in 2019 and 2020—for total and disaggregated food expenditures. We note here that the food groups included in our phone survey differ from the CMIE. It is impossible to separate green leafy vegetables (GLV) and Vitamin A-rich vegetables from the vegetables food group in the CMIE data.

To capture the household reliance on government programs during the pandemic, respondents of the phone survey were asked if they received an additional ration of 5 kgs of wheat or rice and 1 kg of pulses through the PDS. Additionally, they were asked about access to take-home ration (THR) or hot cooked meals from the *aanganwadis* for eligible children/women (if any) and if Rs 1000 was deposited in the bank account of any one member of the household. Furthermore, respondents were asked about their perceptions of how food access, affordability, and availability were impacted during the lockdown. The TARINA phone survey focused on whether local food markets were functional during the pandemic-induced shutdown and the extent to which those markets were able to supply diverse, nutritious foods at affordable prices. The survey asked (a) if the household ability to access and afford foods from rural markets had changed compared to one month preceding the survey (i.e., are they purchasing more/less; perceived changes in availability and prices of food) and (b) if they perceived changes to food availability at the household level. The latter was measured using three self-reported measures where the respondents answer if they felt that the household had (1) lower quantity of food for consumption, (2) lower variety of food, and (3) lesser number of meals per day compared to the days before the lockdown.

#### Food consumption-women's diet diversity

Both rounds of the TARINA primary surveys collected detailed information on women's dietary intake, which was used to construct a women's dietary diversity score (WDDS). The WDDS was calculated based on classifying foods into the following ten groups: grains, pulses, nuts, dairy, meat–fish–poultry or MFP, Dark Green Leafy Vegetables or DGLV, vitamin-A rich fruits and vegetables, other vegetables, and other fruits (FAO, 2016). Women were asked if they consumed food items belonging to each of these food groups in the previous 24 h. A score of 1 was assigned for every food group they consumed, and the DDS is a count of the total food groups consumed in the previous 24 h by the woman. We compared the WDDS from May 2020 to May 2019 to analyze change in women's diets. We also disaggregated the DDS and compared the proportion of women who consumed each food group in the two time periods. The disaggregation allowed us to identify specific food groups (like non-staples) in which the women's consumption was affected more than others (like staples). The aggregate and disaggregated DDS results are compiled for the total sample and each of the four districts.

## Results of the study

### Declining total monthly food expenditures at the state and district levels

The CPHS data depicts a significant decline in household food expenditure during the lockdown. Household monthly food expenditures for March–June in 2020 were significantly lower than for the same period in 2019 at the national, state, and district levels (Table 1). However, there is considerable heterogeneity in expenditure pre-lockdown and decline trends during the lockdown at the national, state, and district levels. Following the lockdown in March, the food expenditure decline in 2020 was pronounced for April and May. In all three states, food expenditures not only recovered but were greater than pre-lockdown (i.e., March 2020) levels by June 2020 at the national and state level. The spending in June 2020 was still below the corresponding expenditures in 2019. We see that the average expenditure for food at the state-level level before the lockdown is slightly below the national average in Bihar and UP and much higher in Odisha. The decline and recovery of expenditure keep with this trend. The magnitude of the decrease in food expenditures during the lockdown is higher in Odisha than the national average and average spending of UP and Bihar.

**Table 1:** Total monthly food expenditures, March–April 2019–2020 (CPHS)

	March (in INR)			April (in INR)			May (in INR)			June (in INR)		
	2019	2020	% Change	2019	2020	% Change	2019	2020	% Change	2019	2020	% Change
All-India	4918.45	4641.57	− 5.63****	4995.94	4153.47	− 16.86****	5061.02	4368.09	− 13.69****	4955.17	4636.89	− 6.42****
State-level												
Bihar	4502.93	4141.38	− 8.02****	4533.83	3797.07	− 16.25****	4630.12	3821.24	− 17.47****	4628.31	4347.01	− 6.08****
Odisha	3607.36	3351.37	− 7.10****	3671.32	2588.18	− 29.50****	3766.85	2832.17	− 24.81****	3782.06	3608.53	− 4.59****
Uttar Pradesh	4397.67	4145.64	− 5.73****	4967.19	4446.80	− 10.48***	5256.57	4602.32	− 12.45****	4399.30	4351.09	− 1.10
District-level												
Munger*	4366.19	3863.15	− 11.52****	4418.75	3816.49	− 13.63****	4601.76	3036.93	− 34.00****	4537.20	3128.67	− 31.04****
Kandhamal	3329.17	2601.38	− 21.86****	3617.34	1601.78	− 55.71****	3597.21	2370.27	− 34.11****	3618.16	2466.22	− 31.84****
Kalahandi	3566.11	2623.75	− 26.43****	3607.76	1596.50	− 55.75****	3580.19	1820.90	− 49.14****	3643.51	2492.50	− 31.60****
Maharajganj	3373.54	3691.67	9.43	3859.17	3562.67	− 7.68	3263.83	6712.33	105.66****	4106.25	3488.53	− 15.04

*Data for Munger is compiled, taking the average for the nearby districts of Bhagalpur, Begusarai, and Khagaria with similar agroclimatic conditions, levels of urbanization, literacy, and household size

The data at the district-level tells a different story. The average food expenditure is below the state and national averages in 2019 and during the lockdown in the four districts. The magnitude of the decline in household food expenditures was greater at the district level than at the state-levels and national level. The decline can be attributed to the selected regions being the lagging districts in economic development within the states. However, unlike the national and state-levels where there is a reversal of declining food expenditures to the pre-pandemic levels (March 2020), at the district-level there is no visible recovery, even by the end of June 2020. The data points to two salient facts. First, as expected, while there was a significant decline in food expenditures in 2020 compared to the same period in 2019, the decline in the less developed districts in UP, Bihar, and Odisha was higher than the state and national averages. Second, while the expenditures recovered to the pre-lockdown levels in March at the state and national levels, the district level expenditures had not recovered. The food expenditure data points out that the households in lagging regions were disproportionately affected by access and availability constraints.

### Declining total monthly expenditures by food groups at the state and district levels

We look at the trends in spending at the household level, disaggregating the food groups, to assess changes in the quality of diets rather than just quantity at the national, state, and district levels using the CPHS data (Table 2). The data allows us to look at the disaggregated household food expenditures on different food groups. At the national level, we see a significant expenditure decline in all food groups in 2020 compared to the same period in 2019 with the largest drop in animal products such as meat, fish and eggs, and fresh and dry fruits. We see an increase in the expenditure on milk and milk-based products at the national level. At the state level, we see considerable variation in patterns of consumption in the three states. Bihar saw a decline in all food groups except vegetables, where it saw an increase in expenditure. In Odisha, there was a large decline in the expenditure on cereals and pulses relative to other states and other food groups. There was also a large increase in expenditure on milk and milk products in Odisha. There is a decline in all food groups other than milk and milk products where there are a slight increase and a substantial increase in the expenditure on vegetables, potatoes, and onions in UP.

**Table 2:** Change in food group-wise monthly food expenditures, May 2019 and 2020 (CPHS)

	Cereal	Pulses	Dry fruits	Milk and milk products	Meat and fish	Eggs	Vegetables	Potatoes and onions	Fruits	Other foods
	% Change	% Change	% Change	% Change	% Change	% Change	% Change	% Change	% Change	% Change
All-India	− 11.72****	− 9.52****	− 36.57****	10.66****	− 47.87****	− 32.55****	− 9.51****	3.53****	− 34.91****	− 20.92****
State-level										
Bihar	− 6.53****	− 0.80	− 77.76****	− 4.65****	− 57.99****	− 47.62****	5.46****	− 5.00****	− 36.77****	− 28.68****
Odisha	− 66.41****	− 31.57****	− 19.68	41.33****	− 33.70****	− 13.26****	− 5.14****	− 12.16****	− 4.38	− 4.48****
Uttar Pradesh	− 15.28****	− 0.50	− 48.96****	1.01	− 77.24****	− 62.48****	20.29****	14.12****	− 33.93****	− 24.09****
District-level										
Munger*	− 33.90****	12.62****	− 88.23****	− 34.56****	− 72.69****	− 64.73****	− 0.36	7.14****	− 50.35****	− 37.93****
Kandhamal	− 58.58****	− 37.47****		111.06****	− 51.26****	− 25.82****	− 29.83****	− 50.65****	− 97.12****	− 23.07****
Kalahandi		− 45.28****		66.23****	− 68.61****	− 36.35****	− 30.34****	− 59.50****		− 17.03**
Maharajganj	455.55****	− 9.13	477.14****	106.37****			191.58****	− 29.21**	− 26.33*	− 8.14

The percentage changes are differences between the average food group-wise expenditure between May 2020 and 2019: ****p < 0.001, ***p < 0.01, **p < 0.05, *p < 0.1

Like the monthly expenditure for food as a whole, the distribution of expenditure across various food groups varies drastically at the state and district levels. In Munger district, expenditures on cereals, pulses, milk and milk products, and other livestock products and fruits declined considerably more than the Bihar state average. In Kandhamal and Kalahandi, the decline in cereals and pulses expenditures was in line with the state-level averages. In contrast, in all other groups other than milk and milk products, the expenditure decline was considerably higher than the Odisha state average. Expenditure on milk and milk-based products was significantly higher in these districts compared to the state average. In Maharajganj there was a significant decline in spending on fruits and vegetables like potatoes and onions.

The increase in milk and milk product expenditure can be seen at the national level and the state and district level in Odisha compared to May 2019. Similarly, there is increased spending on potatoes and onions at the national level and vegetables at the state level in UP and Bihar, and Munger district. The increase may not be surprising and potentially in line with food price inflation, discussed in the next section. While expenditures increase, the volume of food availability could be less. The absence of consumption data for these households prevents us from verifying this from the CPHS dataset. On a similar line of argument, the decline in the expenditure on cereals and pulses could be due to the free PDS provisions in these two food groups. In the absence of data on PDS access for these households, this is hard to verify.

### Affordability of non-staples at the household level: evidence from the phone survey

The decline in household food expenditures evident from the CPHS secondary data is also reflected in our primary data. Using the panel dataset, we compared weekly household food expenditure during the shutdown (May 2020) to food expenditure in 2019. On average, households were spending INR 545.97 per week on food in May 2019. During the lockdown, households saw their weekly food expenditure fall by 11% to INR 482.71 in May 2020. Table 3 shows that DGLVs and fruits rich in micronutrients and eggs, a rich source of protein and nuts and grains, saw a significant decline. Dairy saw an increase (though not significant) in the Kalahandi district. Expenditure on pulses and vegetables increased significantly, as did spending on meats. The increase in dairy and vegetable expenditure corresponds to increases seen at the district, state, and national levels in the CPHS data. Unlike our data, the CPHS data shows an increase in expenditure on pulses. In Odisha, meat, fish, and poultry saw a significant expenditure increase. One reason for this can be that while our survey asked for expenditures in the previous one week, the CPHS data uses monthly data, thereby smoothening week to week fluctuations in food purchase behavior. Another explanation for this is that while our primary data indicates an increase in dairy and vegetable expenditure, it is not significantly different from 2019 levels.

**Table 3:** Percentage change in household weekly food expenditure May 2020 referencing 2019

Food Groups	All Districts	Munger	Kandhamal	Kalahandi	Maharajganj
Grains	− 51.56***	− 9.17	− 92.42****	− 59.20*	− 66.26
Pulses	34.41	− 0.35	58.87**	− 8.25	54.25
Nuts	− 94.98****	− 92.37*	− 36	− 100***	− 100***
Dairy	− 52.67**	− 77.40**	− 17.19	36.29	− 40.49
Meat, fish and poultry	29.08	− 24.84	126.20**	69.70**	− 73.55***
Eggs	− 34.11**	− 91.30****	27.10	17.45	− 100****
DGLV	− 81.63***	− 81.82****	− 91.98****	− 71.69****	− 66.55****
Other vegetables	30.90***	3.11	198.01****	35.60**	− 15.42
Other fruits	− 91.62***	− 97.73****	− 100****	− 90.76****	− 84.62****

Analysis is conducted for 155 households. DGLV stands for dark green leafy vegetables. Significance: ****p < 0.001, ***p < 0.01, **p < 0.05, *p < 0.1

A decline in household incomes and food price inflation during the lockdown substantially changed food expenditures. Cereals like rice and wheat are primarily sourced from their household's own-production and supplemented by the PDS. As the winter wheat harvest was mostly unaffected and the PDS expanded provisions following the nationwide lockdown, the declining expenditures on cereals did not necessarily result in a decline in the household quantity available for consumption. However, households rely on local food markets to meet all other nonstaple food groups' consumption requirements. Seth et al. (2020) compare food commodities' weekly retail prices across major Indian cities for March, April, and May in 2020 against those in 2019. The results for three cities—Patna (in Bihar), Lucknow (in UP), and Bhubaneswar (in Odisha) are in the same program States as our phone survey. They find that retail prices of pulses and vegetables like potatoes and onions were higher during March–May 2020 than the same months one year ago and are in line with the phone survey findings. The significance of price rise for food security is high. With the price rise, the amount of food the household buys for the same amount of money is lower than in earlier periods. While expenditures reduced by 11%, the amount of food households could buy for the same amount previously also reduced.

The increase in food prices immediately after the March lockdown was temporary as the price levels returned to their pre-lockdown levels after May 2020. For commodities such as eggs and tomatoes, prices were lower in 2020 than in 2019 but had stabilized by end-May 2020. Since our phone survey was carried out in the third week of May, it likely reflects the period just before prices returned to pre-lockdown levels. Pulses continued to see an upward trend in prices even after the lockdown ended (Seth et al., 2020). Meat typically is a more costly source of protein in India (Raghunathan et al., 2021)—suggesting that although there was an increase in expenditure on these food groups, it is likely quantities reduced.

### Worsening of dietary quality for women

We compare women's dietary diversity pre and post-lockdown to assess whether there were any changes to women's dietary quality and, if so, by how much and in which specific food groups. To determine the difference, we compare women's WDDS from May 2020 to May 2019 from the same household. On average, women consumed 5.04 food groups in the previous 24 h in 2019, and in 2020 it declined significantly to 4.66 food groups. The WDDS scores declined in all four districts. The declines were most significant in Munger and Kandhamal (Table 4).

**Table 4:** Changes in district-level Women's Diet Diversity Scores (WDDS) in 2020 from 2019

District	WDDS 2019	WDDS 2020	Change
Munger	4.57	3.94	− 0.63**
Maharajganj	4.12	3.90	− 0.21
Kandhamal	5.60	5.08	− 0.52**
Kalahandi	5.89	5.71	− 0.18
Total	5.04	4.66	− 0.38**

*Women's dietary diversity scores are calculated on a scale of 0–10. Munger is a district in Bihar; Maharajganj in Uttar Pradesh; and Kandhamal and Kalahandi in Odisha. Significance: ****p < 0.001, ***p < 0.01, **p < 0.05, *p < 0.1

We disaggregated the WDDS to identify specific food groups that saw a decline among women who reported consuming them in the previous 24-h. The share of women who consumed micronutrient-rich nonstaples like Vitamin A-rich fruits and vegetables and other fruits declined in all districts and significantly in the districts of Odisha during the lockdown (Table 5). Compared to a year before, the proportion of women who consumed other fruits declined by a whopping 80% during the lockdown. There was also a significant decline in the proportion of women who consumed other vegetables in the pooled sample as well as the districts of Munger and Maharajganj. Although not significant, the proportion of women who consumed nuts and dairy products also fell between the two time periods.

**Table 5:** Percentage change in women's weekly consumption in May 2020 referencing 2019

Food Groups	All Districts	Munger	Kandhamal	Kalahandi	Maharajganj
Grains	0.00	0.00	0.00	0.00	0.00
Pulses	2.16	3.23	8.11*	9.10	− 10.53
Nuts	− 6.25	− 100*	100	40	− 60
Dairy	− 8.89	− 42.86	− 40	− 8.33**	35.71
Meat, fish and poultry	5.41	− 72.73**	33.33	76.92**	− 75
Eggs	13.33	− 100*	100	57.14	− 100
DGLV	77.59****	275***	65.22****	25**	400***
Vit-A rich F&V	− 42.53****	− 31.25	− 47.22****	− 37.04**	− 62.5
Other vegetables	− 4.64*	− 11.76*	− 2.5	5.55	− 9.76*
Other fruits	− 79.49****	− 55.56	− 100****	− 96****	− 31.25

Analysis is conducted for 155 households. DGLV stands for dark green leafy vegetables. Significance: ****p < 0.001, ***p < 0.01, **p < 0.05, *p < 0.1

We also find a statistically significant rise in the proportion of women who consumed DGLV (all districts) and pulses (in Kandhamal) post-COVID. While this is encouraging, when looked at in the context of the substantial decline in expenditure on DGLVs (from the previous section), it is plausible that the quantity of intake of DGLV had reduced. Odisha shows the most concerning trends when comparing women's weekly consumption to corresponding household-level expenditures in Table 3. Despite an increase in dairy expenditure at the household level in Kalahandi, we do not see a corresponding significant increase in consumption among women. While there is a significant rise in meat, fish, and poultry expenditures in the Odisha districts, the proportion of women consuming this food group shows significance in Kalahandi and not Kandhamal. While there is an increase in other vegetable expenditure (non-vitamin rich and DGLV) at the household level in the two districts of Odisha, there is no significant increase in women's consumption in Kalahandi, and there is a decline (though not significant) in consumption in Kandhamal. The increase in vegetable expenditure is likely reflected in a higher share of women consuming DGLVs compared to other vegetables.

The respondents reported a similar deterioration in the quantity and quality of preparation of pulses. Some said to have had to halve the amount of dal prepared or prepare thinner dals compared to the pre-lockdown days. Such responses were recorded for other nonstaples like dairy, meat/ fish/ poultry, and eggs. It is likely that not only were fewer women consuming nutrition rich nonstaples during the pandemic but that their consumption amounts declined, as compared to pre-pandemic levels.

The results from the survey suggest a worsening dietary quality for women. A limitation of the study is that we cannot compare women's dietary intakes to other household members. However, there is evidence in the literature to show the lower diet diversity of women relative to other members for the same households. Gupta et al. (2020) show that women’s diet diversity significantly less than that of the households’. Furthermore they note that less than 40% households reported consumption of green leafy vegetables, Vitamin-A rich foods, meats and dairy products. In approximately 20% of these households women’s consumption of these foods was entirely absent, pointing to a significant dietary gap at the intrahousehold level.

Nationally representative data from the NFHS-4 (IIPS, 2017) also points to women being more vulnerable. The NFHS-4 data show fewer women consume fruits, eggs, and meats than men, on average in India and the TARINA states (see Figure A2 in Appendix). In Bihar, 41.5% of men consumed eggs at least once a week compared to 26.8% of women. Around 12% fewer women consumed eggs in UP and fish or chicken in Bihar than men. The proportion of women consuming DGLVs was ten percentage points lower than men in UP. Women's consumption lags men by a similar amount for eggs and dairy products in Odisha (IIPS, 2017). Given the persistent deficit in the nutritional quality of women's diets relative to men, even before the pandemic, it is likely that while diet quality may have worsened for all household members, the magnitude is more significant for women. Harris et al (2020) in their survey of male and female farmers across Assam, Jharkhand, AP and Karnataka find that both, the affordability and consumption of vegetables, fruits and dairy products was significantly reduced amongst women farmers as compared to male farmers.

### Access to safety nets during the lockdown

To assess the food environment change, we look at the self-reported food availability measures from the phone survey. The data across the board suggests that there has been a decline in nutrition security at the household level. Nearly 90% of the respondents claimed that they had less food for consumption, 95% said they had the diversity of food available had reduced, and 25% said that they had to cut down on the meals per day compared to a typical day before the lockdown (Table 6). These estimates are troubling since a fall in the quantity and quality of foods available at the household level can translate into low nutrient intake and absorption, resulting in poor nutritional outcomes eventually.

**Table 6:** Result summary of the telephone survey (*n* = 155)

Variable	Mean (%)
Households where at least one member experienced loss of earnings (%)	96.10
Household received extra PDS ration (5 kg grains + 1 kg pulses)	78.70
Household received Take Home Ration or hot cooked meal from Aanganwadi center	27.70
Household received Direct Benefit Transfer	53.50
Households reported a lower quantity of food available for consumption	87.1
Households reported less variety of food available for consumption	95.5
Households reported fewer meals being consumed per day	25.2
Percentage of households that purchased food group in the last 1 week	
Grains	14.66
Pulses	53.34
Dairy	16.48
Meat, fish, poultry	34.56
Eggs	27.03
Dark green leafy vegetables	30.46
Vitamin-A-rich fruits and vegetables	13.12
Other vegetables	83.77
Other fruits	6.13
Households perceived decrease in market availability of foods	62.6
Households perceived increase in market prices of foods	82.60

Table 6 presents the result summary from the telephone survey on 155 households. About 96% of all households reported at least one family member suffering the loss of earning (Table 6). 72% of eligible households also lost access to take-home rations and hot cooked meals from *aanganwadi* closure. As these centers serve young children and nursing and expecting mothers, this is potentially a significant impact. Despite local rural food markets functioning, albeit, in a limited capacity, four out of five households reported a perceived decline in market level availability of foods and an increase in food prices compared to the previous month. Households predominantly said that fruits and vegetables were the two food groups where reduced availability was the starkest in all districts. In Odisha, MFP, along with fruits and vegetables, suffered a decline. Munger District in Bihar and Kandhamal and Kalahandi in Odisha saw an increase in the prices of rice and pulses, respectively. Seven out of ten households in each district reported not having purchased most food groups across all districts (Table 1).^1^ District-level household purchase behavior is presented in the appendix (Figure A1).

The summary results also show an expansion of PDS provisions in all districts. Among the surveyed households, nearly 80% of them had received additional benefits from the PDS (5 kg grains + 1 kg pulses), and more than 50% received direct benefit transfers. This decline in availability and diversity of food at the household level can be associated with two losses of incomes and/or food price inflation resulting from market disruptions following the lockdown. However, the increased availability of staple grains and pulses through extra rationing by the PDS and, to a lesser extent, the take-home rations and hot cooked meals from the *aanganwadi* center, only to a few households, could have eased some availability and access constraints.

## Discussion and policy recommendations

Using a unique panel dataset, we present evidence on food and nutrition security changes at the household and individual level in rural India following the nationwide lockdown implemented to curb the spread of the virus. We compare household food expenditures and women's dietary intake using data from a phone survey conducted in May 2020 (post lockdown) to data for the same households collected in May 2019. The study attempts to answer two questions: first, the lockdowns impact on household food availability in four districts of UP, Bihar, and Odisha. Second, the lockdown's effect on women's food access and diet diversity in the same households. The two significant findings of this study were, first, that the lockdown had a differential impact on calorie security and nutrition security by way of supply-side disruptions in staple and nonstaple foods. Second, while households overall suffered food availability and accessibility setbacks during the pandemic, it is likely that women were disproportionately affected, pointing to the larger problem of persisting intrahousehold nutritional gaps and the need to address them.

The lockdown has revealed shortcomings of existing safety net programs and the need for change for long-term policy reforms. The lack of diversity in the Public Distribution System, especially concerning nutrition-rich foods, limited outreach of the school feeding program and the aanganwadi system, and inadequate reach of direct benefit transfer are some of the challenges potentially impacting vulnerable groups as women and children. To improve the access and availability of diverse, nutritious foods, direct benefit transfers through which households can assess healthy foods from markets become relevant. Deliberate, intentional focus on gender in the safety-net programs is critical to address the inclusion and disproportionate impact of shocks on women. Well-functioning and deregulated markets also become critical to ensure effective price discovery, reduce supply constraints, and ensure nutritious food availability and affordability to vulnerable groups.

### Household food availability-the importance of food quality and delivery mechanisms

During the lockdown, while the loss of income and temporary food price inflation was responsible for lower expenditures and lower consumption of fruits, vegetables, dairy, and meats, staple grain consumption was smoothened out due to an expansion of the PDS services. The differential impact on the availability of staple versus nonstaple food groups highlights the importance of viewing food security not just as an issue of hunger or calories (that was likely averted/ contained) but more importantly as an issue of ensuring an adequate intake of all micronutrients. The distinction between staples and nonstaples becomes relevant for the design of response mechanisms/ coping strategies introduced by governments in response to exogenous shocks such as a pandemic. From the standpoint of health outcomes, policies need to view calorie hunger as distinct from micronutrient hunger.

The government was quick to initiate measures in response to the loss of income and disruptions to food markets that followed the nationwide shutdown in March 2020, in the form of expanding the PDS and roll-out of direct benefit transfers. Households in our survey locations – Bihar, UP, Odisha—source rice and wheat primarily from their own-production and supplementing it with ration from the PDS. Research suggests that PDS is known to shield the beneficiaries from price fluctuations in food grains, ensure food security, and attain minimum nutritional standards (Gadenne et al., 2017; Kumar et al., 2020). Seth et al. (2020) conclude that cereals' supply was largely uninterrupted during the lockdown, and their prices were quick to stabilize to pre-pandemic levels. They attribute this primarily to large buffer stocks released through the NFSA while government procurement of wheat continued—both factors reflect India's long-standing cereal-centric policies.

### Expanding the food groups in the PDS beyond wheat and rice

While the provisioning of cereals and pulses through the PDS addresses food security (i.e., ensuring adequate calories), it is not a sufficient response for micronutrient malnutrition that likely worsened during the pandemic. However, the PDS can be leveraged to meet India's nutrition security goals partially. The PDS can be utilized to deliver nutrient-dense non-coarse cereals such as various millets, sorghum among others and more pulses than it currently distributes. Integrating nutrition-rich foods in the PDS food basket is the first step to ensure that these foods reach intended beneficiaries. Our phone survey indicates that even though pulses—an essential source of protein in the Indian diet—were disbursed through the PDS during the pandemic, it appears that they did not reach beneficiaries as much as cereals. While almost all the households received 5 kgs of wheat or rice, most had not received any pulses. In addition to the PDS, the food provided through aanganwadi centers (either as hot cooked meals or take-home rations) can be expanded to ensure households and vulnerable members within the households have access to micronutrient-rich food. More economically backward districts have more necessity for a diverse PDS system. Strengthening delivery systems and plugging leakages in the system can ensure higher reach.

### Conditional and unconditional direct benefit transfers, functioning markets, and diet diversity

The PDS infrastructure is not adequate for the distribution of perishable nonstaple produce. The high cost of public storage, transportation, and distribution make their distribution through safety-nets untenable. Increasing accessibility to nonstaples can occur through conditional or unconditional direct benefit transfer (DBT) or cash transfers. DBTs can shield households from shocks of income loss and aid food access where markets function. In our survey, 53% of households stated that they were recipients of unconditional DBT. While the direct impact of DBT on consumption change was not ascertained, cash flow increases household options of purchasing foods not made available through the PDS, which is often nonstaples. MNREGA, India's employment guarantee scheme, is a form of conditional case transfer, where individuals can demand 100 days of paid labor from the state. Wage labor in times of low employment opportunities can supplement household incomes, allowing for higher purchasing power. Since our phone survey, the government has announced new measures like increasing the minimum wage in the MNREGA.

During the pandemic, supply chain breakdown for nonstaples such as fruits and vegetables increased prices and is expected to have shifted consumer demand further towards cereals (Seth et al., 2020). Mahajan and Tomar (2021) however draw an important distinction between the impact of market disruptions on perishables and non-perishables. They note that the farm-gate quantities of perishables remained less than their pre-lockdown levels, even as the quantities of non-perishables bounced back by the time the (first) lockdown was lifted in April. The mandi arrivals for fruits and vegetables fell by 42% for commodities that are produced further away from retail centers, suggesting that long-distance freight disruptions were, atleast partly, behind the decline. Removing bottlenecks in food supply chains for nonstaple commodities will be crucial to maintaining stability in commodity prices and improved availability. ICTs and infrastructure such as cold chains and removing market restrictions that limit agricultural produce movement between markets and state boundaries can enable better distribution and price discovery in perishable commodities. Linking farms with buyers through vertical coordination can also reduce supply constraints and improve the availability of nonstaples. Finally, commercialization of small farms and ensuring access to capital and credit can support the diversification of the food system away from staple grains towards horticulture crops improving incomes and availability of nutrient-rich crops in markets (Kumar et al., 2020; Padhee & Pingali, 2020). India risks a reversal of gains made in combatting malnutrition in the past decade if agricultural supply chains for non-staples cannot supply the rising demand for nutrient-rich, high nutritional food groups.

### Intrahousehold allocation of food-remedying the gender disadvantage

The phone survey evidence points to women's access to quality food disproportionately affected. Compared to the baseline, we see a decline in women consuming vitamin A-rich fruits and vegetables, other fruits, and other vegetables by 42%, 79%, and 5%, respectively. One limitation of the study is that while the data indicates that women's dietary diversity worsened during the pandemic, we do not have data to compare the decline to other household members, for example, the men or the children. However, recent evidence suggests that women face a dietary gap compared to other household members (Gupta et al., 2020). Furthermore, NFHS-4 data also indicate that the proportion of women who consumed nonstaples like fruits, eggs, and meats was lower than men (see Appendix). The unaffordability of nonstaples at the household level and reduced diversity of diet among women suggests that the pandemic is likely to have adversely affected household level diet diversity, with a disproportionate impact on women.

Both rounds of the primary survey data were conducted in the month of May that corresponds to the lean season in the agricultural cycle in our locations. Even in the absence of exogenous shocks to the food system it has been shown that women experienced a greater decline in body weight (of 2–3%) as compared to men, between the lean and planting seasons in India (Rao & Raju, 2020). This has been largely attributed to a reduced intake of food as employment levels are low at this time of the year and households reserves of food and cash are nearly exhausted. We therefore posit that the shocks to household income and disruption of food markets that followed the nationwide lockdown in India are not only likely to have worsened nutritional outcomes for women, but that the gendered differences are likely to have magnified.

### Importance of aanganwadi centers of women's nutrition

Women's diets, particularly for pregnant and lactating mothers, would also have been adversely affected by the aanganwadi center closures. Many aanganwadi centers that distribute take-home rations for new mothers remained closed from March until November 2020. Less than 30% of the households had received take-home rations or hot cooked meals from *aanganwadis* at the time of the phone survey in May 2020. In contrast, in 2019 nearly 40% of households received take-home rations during the same month. In some states, take-home rations were provided to eligible beneficiaries in their homes.^2^ While this would have buffered the otherwise adverse impact of the lockdown, it is unknown if the rations provided at the doorstep reached all beneficiaries or not. The baseline from 2019 points out that in non-pandemic times too, only 40% of eligible households had access to take-home rations. Expanding the reach of *aanganwadi* centers will play a critical role in improving the health outcomes of young children and lactating mothers.

### Safety-nets and rectifying household level allocations

While the change in diet diversity scores does not tell us much about the quantity or quality of food consumed, it does shed light on which food groups were consumed by the women of the household. The changes in diet diversity from the phone survey, while apparent in certain food groups such as fresh foods, did expose some nuanced changes in food preparation behavior to adapt to changes in availability. Our results indicate that the proportion of women who consumed pulses in 2020 was the same compared to 2019. However, during the phone survey, women shared important changes related to the preparation and consumption of pulses in their households during the lockdown. These ranged from having to halve the quantity of dal per meal and preparing a thinner/diluted version of the dal compared to the pre-lockdown days. Such responses were recorded for other nonstaples like dairy, meat/fish/poultry, and eggs. The role of safety nets such as direct benefit transfer and diversified PDS, while critical to household-level food security, if inadequate, will not reach the women of the household. Adequate provision of PDS goods and adequate direct benefit transfer is critical for women to benefit.

Other works have discussed the impact of the pandemic on women in terms of loss of employment, increasing burden of unpaid care work, access to health services, especially prenatal, neonatal and postnatal, preventive messaging, and increased risk of gender-based violence (Deshpande, 2020; Kumar et al., 2019; Quisumbing et al., 2020; UN, 2020). The pandemic's impact on women's health and nutrition through access to nutritious food is another important dimension that needs more research. Economic, environmental, and health shocks in the form of pandemics affect economically vulnerable populations globally. Marginal groups and women face social and economic discrimination in their everyday lives at the household and societal levels, which is aggravated in crisis and scarcity. Accounting and addressing this at a policy and developmental program implementation level is critical. Designing safety nets and rural development programs with a specific gendered focus is vital to address women's disadvantage in everyday lives and resilience in times of shock.

This study has two limitations—first, the small sample size of households used in the study and two, the lack of a comparison of women's dietary diversity to other family members. While our panel data is unique allowing a comparison of food and nutrition security outcomes before and during the lockdown, the primary data's sample size limits its external validity. While the telephone survey allowed for a quick roll-out, resource limitations narrowed a broader survey scope. By comparing household monthly food expenditures for the same months in 2019 and 2020 with the CPHS data, we try to validate our findings to address some external validity concerns. The study is also limited in accounting for the pandemic's gendered impact relative to other household members. While our data indicate that women's dietary diversity worsened during the lockdown, we do not have data to compare it to the dietary diversity of other household members, such as the men or the children. We use evidence from previous studies and the NFHS-4 data to emphasize the disadvantage of women in food allocation at the household level as a starting point.

It is interesting to note that at the state-level, household food expenditures had returned to their pre-pandemic levels by June of 2020. Simultaneously, prices of non-staples too had begun to stabilize across several big and small cities across the country (Seth et al., 2020). Taken together, this suggests that the supply-side bottlenecks and associated food price inflation were temporary at best.

By June of 2020, prices had declined, and demand for food increased; both factors are likely to have impacted food and nutrition security at the household and individual level. We are limited by data unavailability for June 2020 to identify if consumption did pick up or if there continued to be a lag in consumption, particularly for women. Recent evidence from the World Bank’s Rural Shock Survey suggests that even as much as four months (September) after the lockdown was removed nearly 50% households in Bihar and 40% households in UP—two of our program states—continued to report a decline in food intake (Murali & Maiorano, 2021). This study however is limited by data in drawing comparisons to a pre-COVID baseline. Therefore it is not clear if food insecurity was present in these locations even pre-COVID, and if so what its magnitude was. On the market front Lowe et al (2021) show that mandi arrivals having picked up by June 2020 saw a decline in August and September in that they were both, lower than June 2020 as well as corresponding levels in 2018 and 2019. Therefore it appears that the post-lockdown recovery was short-term.

Although the focus of this paper has been restricted to lagging states of India, like Bihar, Uttar Pradesh and Odisha, the effect of the lockdown was felt across the country, but differently. It appears that farmers in high-income states, like Haryana were buffered from the adverse shock mainly due to resilient market infrastructure that continued public procurement of food grains during the pandemic. However, as consumers, agricultural households in Haryana faced food shortages due to low availability of foods in local markets (Ceballos et al., 2020). In contrast, smallholder farmers in Odisha, overcame disruptions in local food markets by relying on staple and nonstaple food groups they grew at the household level to meet consumption needs during the lockdown, even with production and marketing disruptions due to the low availability of mechanization and an inefficient procurement system (Ceballos et al., 2020). Similarly (Cariappa et al., 2021) note that while high performing states faced labor shortages due to reverse migration from states like Punjab and Haryana while the poorer states were most affected by disruptions in agricultural input supply. However they find a reduction in food consumption across the country, irrespective of the incidence of COVID-19. Nearly 70% of respondents perceived an increase in food prices and 40% reported income shocks, and almost 80% said to have reduced their food consumption. Harris et al (2020) find that food and nutrition security shocks were felt more strongly in low income states like Assam and Jharkhand where 50% and 90% of the respondents reported a decline in food consumption. This was higher than the 30% households that reported the same response to the pandemic in middle income states like Andhra Pradesh and Karnataka. Across states, consumption declined for fruits and meats, eggs, poultry.

It is essential to note that the pandemic-induced shock is different from natural disaster-induced and other economic shocks such as drought, floods, and price fluctuations that rural communities and the agricultural sector is periodically exposed to. The pandemic-induced shock was different in three ways. First, weather-related and cyclical price shocks are seasonal, whereas the pandemic, while initially was predicted to last a short while, has raged on, with the worst effects coming a year later. Second, unlike weather-related shocks that can be regional or price shocks that can be commodity-specific, the pandemic impacted all economic sectors and affected the whole nation. Third, the pandemic was an unfamiliar shock, while the other shocks are recurring and rural communities have coping or mitigating mechanisms to deals with them. The unpredictable, unfamiliar, and pan-economic nature of the pandemic shock has impacted the potential to identify and utilize alternative options otherwise available such as off-farm employment, urban migration, and remittance earnings. Reverse migration from urban to rural areas meant a sudden and unexpected halt in earnings and remittances that were safety nets in weather-related and economic shocks.

The COVID-19 induced lockdown has impacted nutrition security due to food value chain disruptions, influencing food access and availability. We infer that the disruptions have been felt disproportionately even at the household level. This paper’s primary and secondary data point to a decline in food expenditure and in women’s dietary diversity from May 2019 to May 2020. Consumption of nutritious non-staple food groups such as meat, eggs, vegetables, and fruits showed the most significant decline and may have long-term health repercussions. Our findings contribute to the growing body of evidence of women’s disproportionate vulnerability to shocks*.*

## Figures and Tables

**Fig. 1:**
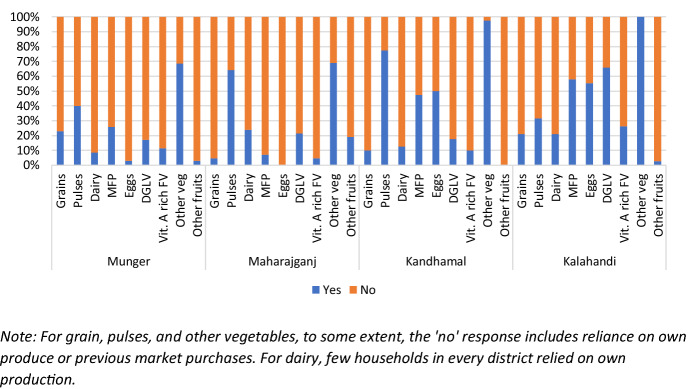
Food groups purchased by households in last one week, May 2020

**Fig. 2:**
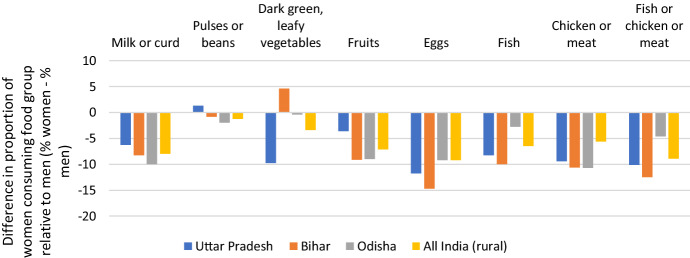
Difference in consumption of food groups by women relative to men, atleast once a week (NFHS-4)

## Data Availability

Data can be made available upon request.

## Appendix

See Figs. 1 and 2.

## Footnotes

1 Similar evidence pointing to the inability of households to access and afford food insufficient quantity and variety has been reported from Haryana and Odisha (Ceballos et al., 2020).

2 World Bank: https://www.worldbank.org/en/news/feature/2020/04/27/covid19-coronavirus-india-response-health-undernutrition-anganwadi-workers-healthcare#:~:text=In%20normal%20times%2C%20India's%20million,the%20battle%20against%20COVID%2D19.&text=%22In%20India%2C%20the%20anganwadi%20system,and%20now%20against%20COVID%2D19.

## Code availability

Code can be made available upon request.
